# Oestrogen inhibits resveratrol-induced post-translational modification of p53 and apoptosis in breast cancer cells

**DOI:** 10.1038/sj.bjc.6601902

**Published:** 2004-06-08

**Authors:** S Zhang, H J Cao, F B Davis, H-Y Tang, P J Davis, H-Y Lin

**Affiliations:** 1 The Ordway Research Institute, 150 New Scotland Avenue, Albany, NY 12208, USA; 2 The Research Service, Stratton Veterans Administration Medical Center, 113 Holland Avenue, Albany, NY 12208, USA; 3 The Wadsworth Center, New York State Department of Health, Empire State Plaza, Albany, NY 12201, USA

**Keywords:** breast cancer, MCF-7 cells, p53, resveratrol, oestrogen, apoptosis, mitogen-activated protein kinase

## Abstract

Resveratrol, a naturally occurring stilbene, induced apoptosis in human breast cancer MCF-7 cells. The mechanism of this effect was dependent on mitogen-activated protein kinase (MAPK, ERK1/2) activation and was associated with serine phosphorylation and acetylation of p53. Treatment of MCF-7 cells with resveratrol in the presence of 17*β*-oestradiol (E_2_) further enhanced MAPK activation, but E_2_ blocked resveratrol-induced apoptosis, as measured by nucleosome ELISA and DNA fragmentation assays. E_2_ inhibited resveratrol-stimulated phosphorylation of serines 15, 20 and 392 of p53 and acetylation of p53 in a concentration- and time-dependent manner. These effects of E_2_ on resveratrol action were blocked by ICI 182,780 (ICI), an inhibitor of the nuclear oestrogen receptor-*α* (ER). ICI 182,780 did not block the actions of resveratrol, alone. Electrophoretic mobility studies of p53 binding to DNA and of *p21* expression indicated that E_2_ inhibited resveratrol-induced, p53-directed transcriptional activity. These results suggest that E_2_ inhibits p53-dependent apoptosis in MCF-7 cells by interfering with post-translational modifications of p53 which are essential for p53-dependent DNA binding and consequent stimulation of apoptotic pathways. These studies provide insight into the complex pathways by which apoptosis is induced by resveratrol in E_2_-depleted and -repleted environments.

Resveratrol is a phytoalexin that occurs naturally in grape skin and several medicinal plants (Jeanet *et al*, 1991; Lu and Serrero, 1999). It has anticancer (Jang *et al*, 1997) and other biologic properties (Pace-Asciak *et al*, 1995). The mechanism of the antitumour effect of resveratrol is not well understood, but in some tumour cell models induction of apoptosis is involved (Clement *et al*, 1998; Huang *et al*, 1999; She *et al*, 2001). We have demonstrated resveratrol-induced apoptosis in four thyroid cancer cell lines (Shih *et al*, 2002) and two prostate cancer cell lines (Lin *et al*, 2002). Resveratrol has been shown to exert mixed oestrogen agonist/antagonist activities in mammary cancer cell cultures, but in the presence of 17*β*-oestradiol (oestradiol, E_2_), resveratrol may function as an antioestrogen (Bhat *et al*, 2001), antagonising E_2_ activities. In this study, we have explored the effects of resveratrol and E_2_, both separately and together, in breast cancer (MCF-7) cells, and show that E_2_ inhibits resveratrol-induced apoptosis in these cells.

p53 is an oncogene suppressor protein that is present at low levels in normal cells (Higashimoto *et al*, 2000). In response to a variety of stresses, including DNA damage (Higashimoto *et al*, 2000), levels of cellular p53 protein rise; this increase in p53 results at least in part from a post-translational mechanism that stabilises the protein (Ashcroft and Vousden, 1999). p53 can adopt two forms *in vitro*, a latent form that binds naked DNA poorly and an active form that binds to DNA well. Conversion of the latent form to the active form is thought to occur by an allosteric mechanism conditioned by phosphorylation and acetylation (Sakaguchi *et al*, 1998; Liu *et al*, 1999; Prives and Manley, 2001). Serine phosphorylation of wild-type p53 at different sites has different biological consequences (Agarwal *et al*, 1998). We and others have shown that resveratrol-induced serine phosphorylation of p53 in various cancer cells is essential to resveratrol-induced apoptosis (She *et al*, 2001; Lin *et al*, 2002; Shih *et al*, 2002). Acetylation of p53 occurs in the wake of serine phosphorylation and is essential for p53-dependent gene expression (Berlev *et al*, 2001). In this report, we show that although both E_2_ and resveratrol stimulate mitogen-activated protein kinase (MAPK; ERK1/2) activity in an additive manner in MCF-7 breast cancer cells, E_2_ *inhibits* resveratrol-induced serine phosphorylation and acetylation of p53, as well as oligonucleotide binding of p53. As a result, resveratrol-induced p53-dependent *p21* gene expression and apoptosis are inhibited by E_2_ in these breast cancer cells.

## MATERIALS AND METHODS

### Cell lines and reagents

The MCF-7 human breast cancer cells were provided by Dr J Bennett (Albany Medical College, Albany, NY, USA). Cell lines were maintained for study in DMEM supplemented with 10% foetal bovine serum (FBS), in a 5% CO_2_/95% O_2_ incubator at 37°C. Cells were placed in 0.25% serum-supplemented medium for 2 days prior to treatment. The serum used for these studies was previously depleted of oestradiol by ion exchange resin, resulting in a total E_2_ concentration in undiluted serum of <10^−11^ M (Dr Z Cao, New York State Department of Health, Albany, NY, USA, personal communication). Oestradiol and resveratrol were obtained from the Sigma Chemical Company (St Louis, MO, USA), and ICI 182,780 was obtained from Tocris Cookson Inc. (Ellisville, MO, USA).

### Cell fractionation

Fractionation and preparation of nucleoproteins was carried out according to our previously reported methods (Shih *et al*, 2001; Lin *et al*, 2002). Nuclear extracts were prepared by resuspension of the crude nuclei in hypotonic buffer with 420 mM NaCl, 20% glycerol at 4°C with rocking for 1 h, and the supernatants were collected after subsequent centrifugation at 4°C and 13 000 rpm for 10 min.

### Immunoblotting

**Figure 1:**
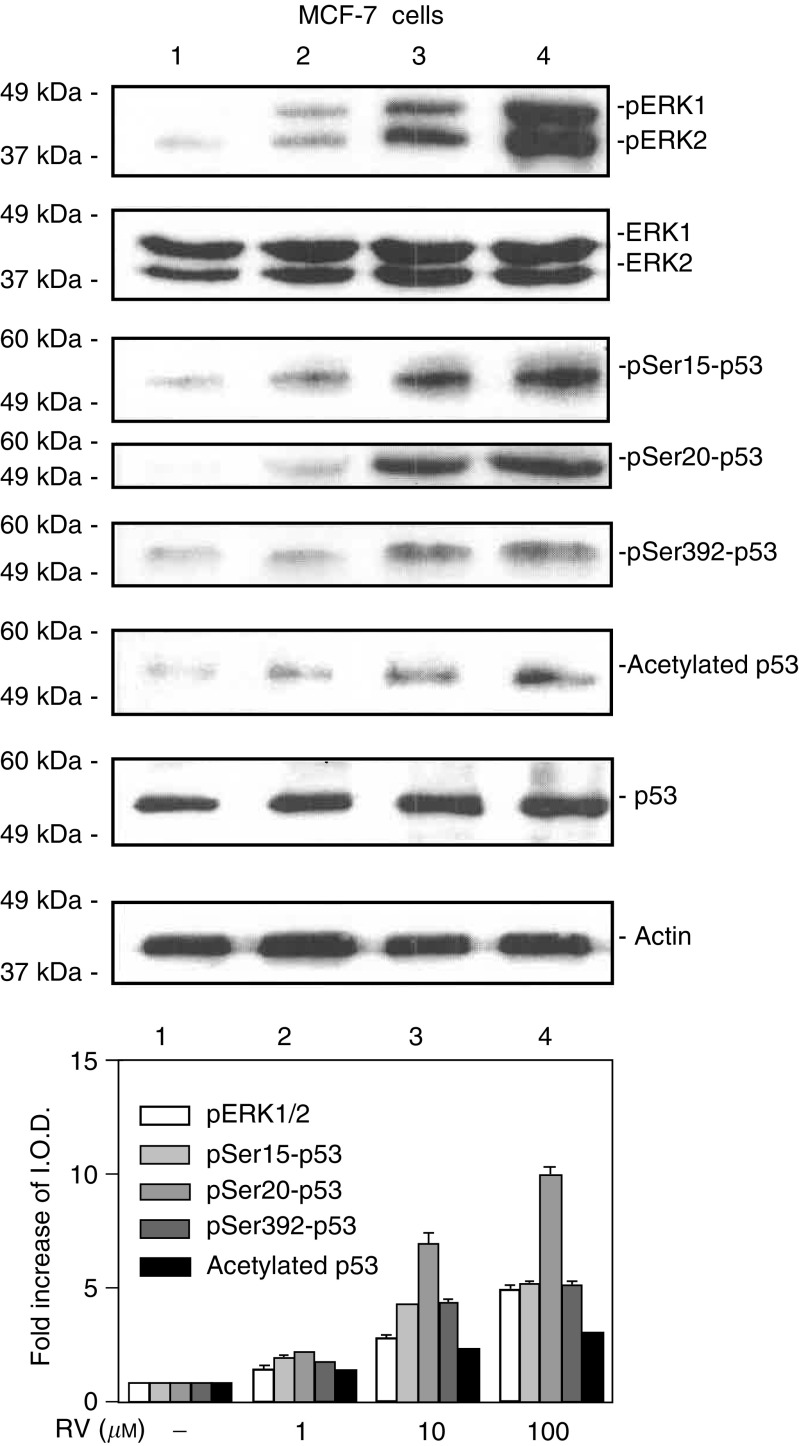
Resveratrol treatment of MCF-7 cells leads to phosphorylation (activation) of MAPK, serine phosphorylation of p53 and acetylation of p53 in MCF-7 breast cancer cells. Cells were treated with 1–100 *μ*M resveratrol (RV) for 4 h. Nuclear proteins were separated by electrophoresis, and immunoblots performed with selected antibodies. Mitogen-activated protein kinase activation, shown as appearance in nuclei of nuclear phosphorylated ERK1 and ERK2 (pERK1 and 2), and phosphorylation of serines 15, 20 and 392 of p53, as well as acetylation of p53, were induced by resveratrol in a concentration-dependent manner (lanes 2–4). Increases in phosphorylation of ERK1/2 and acetylation and phosphorylation of p53 with 1–100 *μ*M RV were each significant at *P*<0.001 by one-way analysis of variance in three similar experiments. The graph below shows the changes in phosphorylation of ERK1/2 and p53, and acetylation of p53, normalized to a value of 1 in control samples, in three experiments. Means ± s.e.m. are shown. Immunoblots of total nuclear ERKs 1 and 2 (second panel from top) are consistent with activation (phosphorylation) and nuclear accumulation of these proteins, and a slight increase in total nuclear p53 is consistent with the resveratrol effect which we have previously described (Lin *et al*, 2002; Shih *et al*, 2002). Actin immunoblots serve as loading controls in this and subsequent figures. Immunoblots in all figures are representative of three or more experiments. Molecular weights of the proteins shown are as follows: pERK1, 44 kDa; pERK2, 42 kDa; p53, 53 kDa; actin, 43 kDa. (I.O.D., integrated optical density).

**Figure 2:**
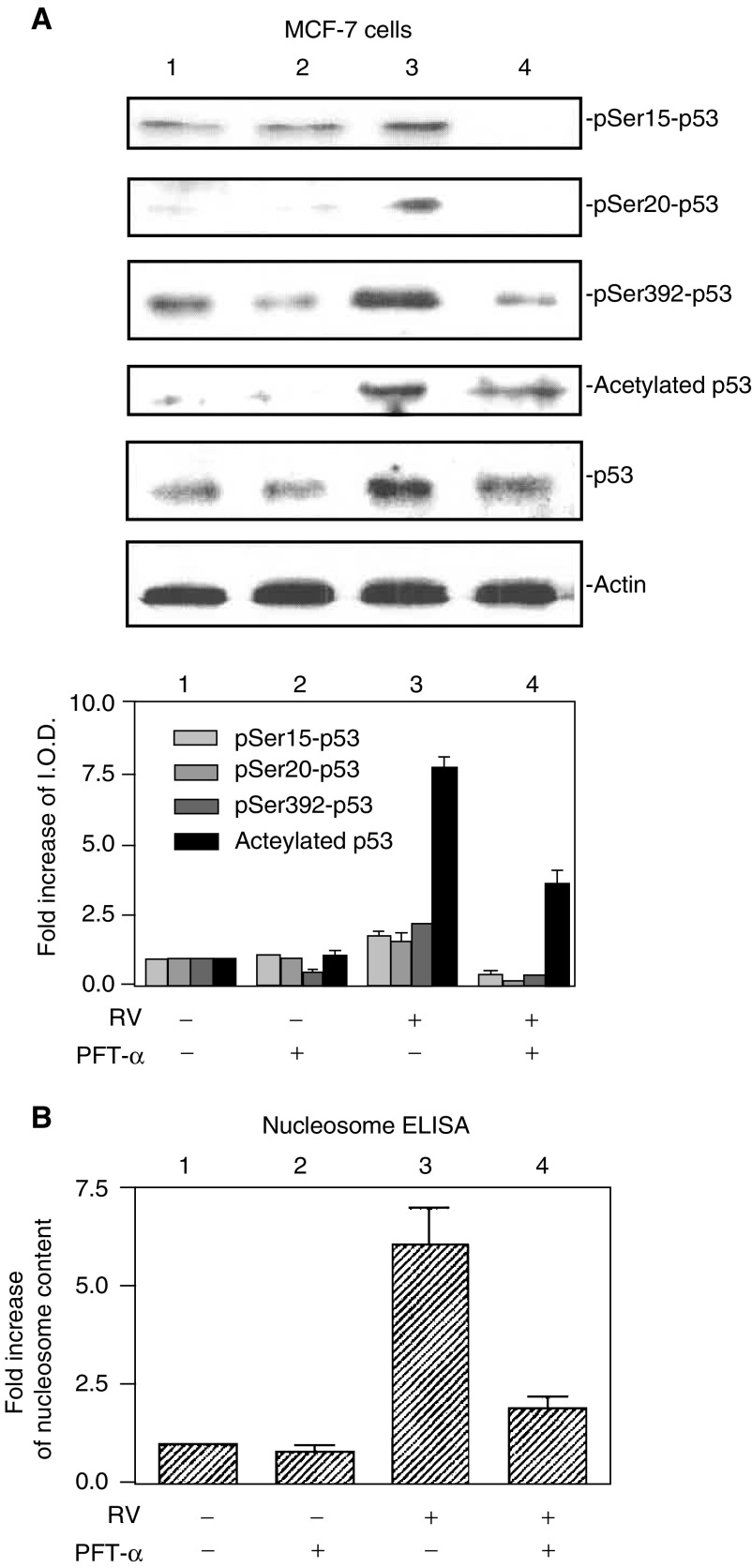
Activation of p53 and apoptosis induced by resveratrol is inhibited by pifithrin-*α* in MCF-7 cells. (**A**) Cells were treated with 10 *μ*M resveratrol (RV) for 4 h in the presence or absence of 20 *μ*M pifithrin-*α* (PFT-*α*), a specific p53 inhibitor. As shown by immunoblots of nuclear fractions from a representative experiment above, and the accompanying graph below, resveratrol caused nuclear accumulation, serine phosphorylation and acetylation of p53 (*P*<0.05 comparing lanes 1 and 3 for each parameter). Treatment with PFT-*α* resulted in decreased phosphorylation and acetylation of p53 (*P*<0.05 for all parameters, comparing lanes 3 and 4). Molecular weight markers in this figure are not shown, but are similar to those shown in Figure 1. (**B**) Cells were treated with 10 *μ*M resveratrol for 24 h in the presence or absence of 20 *μ*M PFT-*α*. Apoptosis, shown by an increase in nucleosome content determined by ELISA, occurred with resveratrol treatment (*P*<0.001, comparing lanes 1 and 3. Pifithrin-*α* had no effect alone, but significantly blocked resveratrol-induced apoptosis (comparing lanes 3 and 4, *P*<0.005). Means±s.e.m. of six experiments are shown.

The techniques are standard and have been previously described (Shih *et al*, 2001; Lin *et al*, 2002). Nucleoproteins were separated on discontinuous SDS–PAGE (9% gels), and then transferred by electroblotting to Immobilon membranes (Millipore, Bedford, MA, USA). After blocking with 5% milk in Tris-buffered saline containing 0.1% Tween, the membranes were incubated overnight with polyclonal antibodies to phosphorylated ERK1/2, or serine 6- (ser6-), ser15-, ser20- or ser392-phosphorylated p53 (Cell Signaling, Beverly, MA, USA). A polyclonal antibody to acetylated p53 was from Upstate Biotechnology (Lake Placid, NY, USA). The secondary antibody was goat anti-rabbit IgG (1 : 1000, Dako, Carpenteria, CA, USA). Immunoreactive proteins were detected by chemiluminescence (Amersham Life Science, Arlington Heights, IL, USA). All immunoblots were scanned and optical densities of bands were measured. Immunoblots shown in the figures are representative of three experiments. Statistical significance of changes in phosphorylation of ERKs 1 and 2, and of p53, as well as p53 acetylation, was calculated by one-way analysis of variance on data from three experiments. Molecular weight markers are included in Figure 1

for reference; band molecular weights are similar to those in Figure 1 in Figures 2

, 3

**Figure 3:**
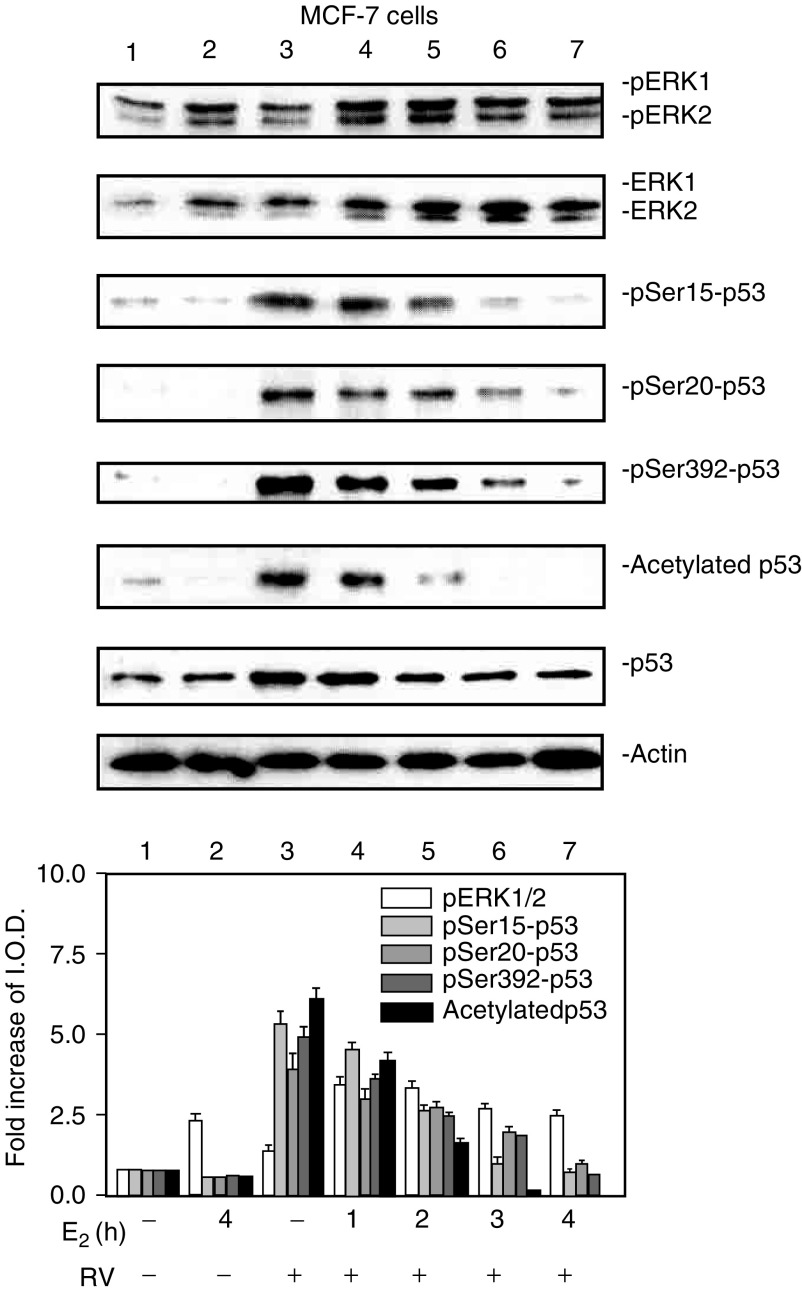
E_2_ inhibition of resveratrol-induced activation of MAPK and p53 is time dependent. MCF-7 cells were treated with E_2_ (10^−9^ M) alone for 4 h or for different time periods (0–4 h) with resveratrol (RV, 10 *μ*M, 4 h), after which nuclear fractions were prepared from each sample. This series of immunoblots, representative of three experiments, indicates that minimal activation of ERK1/2 by resveratrol (top panel, lane 3) was enhanced by 1–2 h incubation with E_2_ (lanes 4 and 5). A longer incubation with E_2_ caused a time-dependent reduction in ERK1/2 phosphorylation (lanes 4–7, *P*<0.05). Resveratrol induced phosphorylation of serines 15, 20 and 392 of p53 (pSer15-, pSer20- and pSer392-p53), and acetylation of p53 (lane 3 in each panel). However, these effects of resveratrol on p53 post-translational modification were progressively inhibited by coincubation with E_2_ for 1–4 h. The reductions in resveratrol-induced serine-15, serine-20, and serine-392 phosphorylation and acetylation of p53 by E_2_ were significant at *P*<0.003 or less. Molecular weight markers in this figure are not shown, but are similar to those shown in Figure 1.

, 4

**Figure 4:**
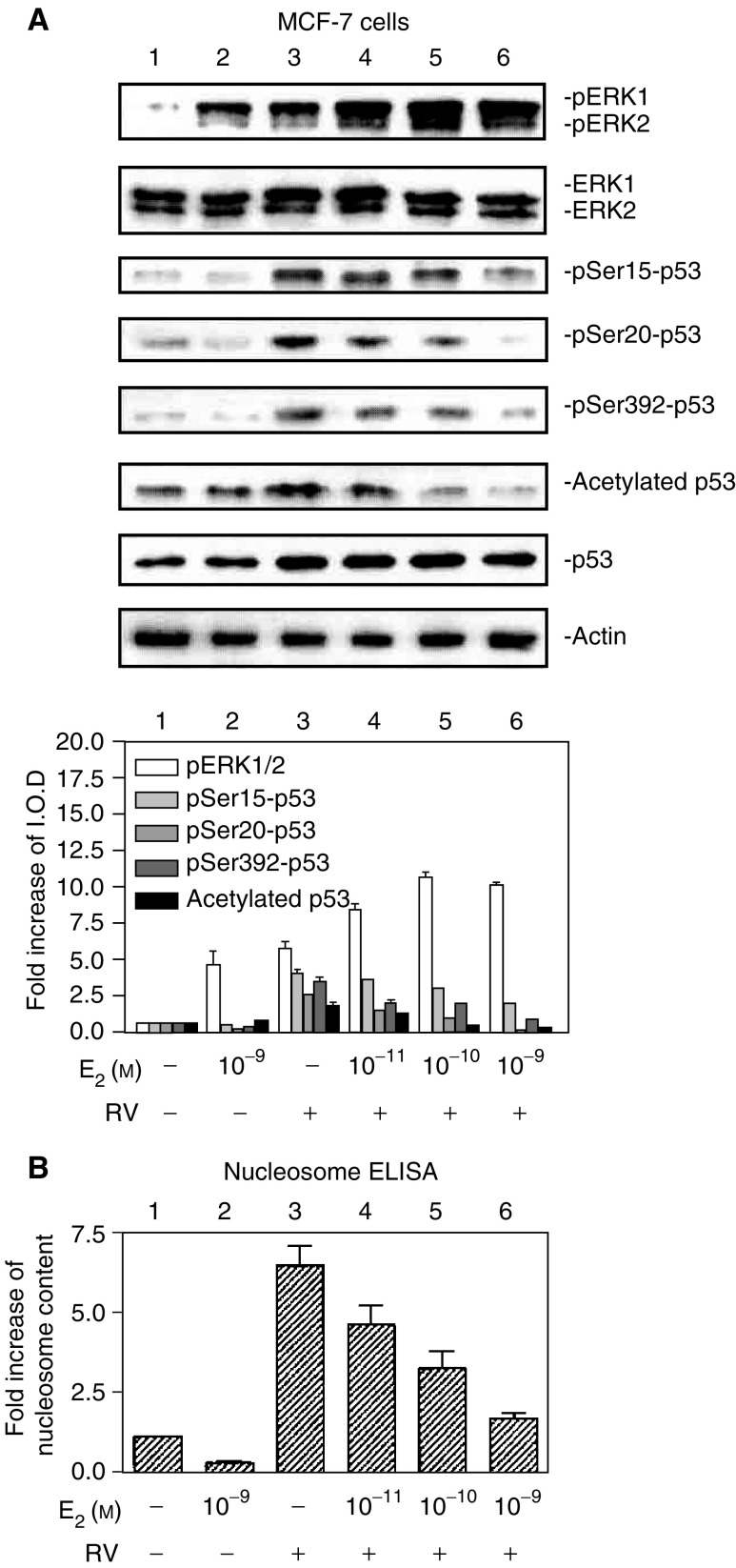
E_2_ inhibits resveratrol-induced p53 phosphorylation and acetylation, and subsequent apoptosis, in a concentration-dependent manner. (**A**) MCF-7 cells were treated with E_2_ (10^−11^–10^−9^ M) with or without resveratrol (RV, 10 *μ*M) for 4 h. Immunoblots of nuclear fractions from this representative experiment show that resveratrol-induced MAPK activation (pERK1/2, top panel, lane 3) was enhanced by 10^−11^–10^−9^ M E_2_ (lanes 3–6; *P*<0.005). However, resveratrol-induced ser15, ser20 and ser392 phosphorylation of p53, as well as acetylation of p53, was inhibited by E_2_ in a dose-dependent manner (*P*<0.001, lanes 3–6 for all these parameters), with the most marked inhibition occurring at 10^−9^ M E_2_ (lane 6). Molecular weight markers in this figure are not shown, but are similar to those shown in Figure 1. (**B**) This graph shows the extent of apoptosis, measured by nucleosome ELISA, in MCF-7 cells treated with E_2_ (10^−11^–10^−9^ M) with or without resveratrol (RV, 10 *μ*M) for 24 h. Resveratrol alone caused apoptosis (lane 3, *P*<0.001). E_2_ alone did not induce apoptosis, but the hormone inhibited nucleosome formation induced by resveratrol in a dose-dependent manner (*P*<0.001). Means±s.e.m. of five experiments are shown.

and 5

**Figure 5:**
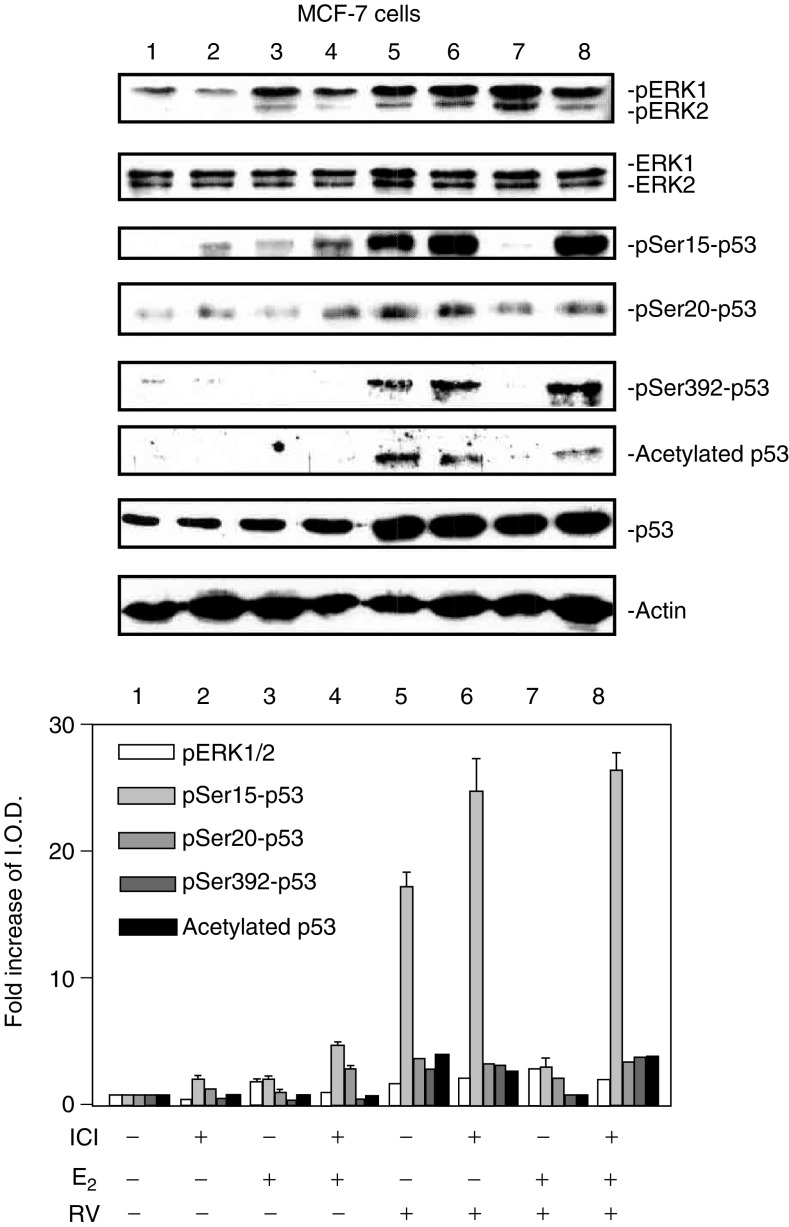
ICI 182,780 (ICI) inhibits the effects of E_2_ on resveratrol-induced activation of MAPK and p53. MCF-7 cells were pretreated with 3 nM ICI or diluent for 30 min, after which they were treated with 10^−9^ M E_2_ and/or 10 *μ*M resveratrol for 4 h along with continued 3 nM ICI or diluent as in the pretreatment period. Results shown are representative of three experiments. Immunoblots of nuclear fractions show that ICI minimally inhibited E_2_-induced MAPK activation (comparing lanes 3 and 4), but did not inhibit resveratrol-induced MAPK activation (lanes 5 and 6). ICI 182,780 did enhance resveratrol-induced ser15-p53 phosphorylation, although this effect was not statistically significant. The additive effect on MAPK activation of E_2_ and resveratrol was reduced by ICI (*P*<0.05, lane 7 *vs* lane 8), and the inhibitory effect of E_2_ on resveratrol-induced serine phosphorylation and acetylation of p53 was partially reversed by ICI treatment (*P*<0.02 for each parameter, comparing lanes 7 and 8). Molecular weight markers in this figure are not shown, but are similar to those shown in Figure 1.

.

### Electrophoretic mobility gel shift assay (EMSA)

Nuclear extracts (10 *μ*g protein) were incubated in a 25 *μ*l total reaction volume that contained 10 mM TRIS, pH 7.5, 50 mM NaCl, 1.0 mM MgCl_2_, 0.5 mM EDTA, 0.5 mM DTT, 4% glycerol and 0.05 *μ*g *μ*l^−1^ poly(dI-dC) (Promega, Madison, WI, USA). A p53-binding oligonucleotide (Santa Cruz, Santa Cruz, CA, USA), labelled with [^32^P] by T4 polynucleotide kinase (Promega), was added to the total reaction mixture, which was then incubated for 20 min at room temperature. A control nonreactive SP-1 oligonucleotide (Promega) was prepared in the same manner. Samples were loaded on 4% polyacrylamide gels in low ionic strength buffer (22.3 mM Tris, 22.2 mM borate, 0.5 mM EDTA) and run with cooling at 15 V cm^−1^. The gels were then dried, exposed to X-ray film and autoradiographs were analysed.

### RT–polymerase chain reaction (PCR)

Total RNA was isolated as described previously (Shih *et al*, 2002). First-strand complementary DNAs were synthesised from 1 mg of total RNA using oligo-deoxythymidine and AMV Reverse Transcriptase (Promega). First-strand cDNA templates were amplified for *p21* and *GAPDH* by PCR using a hot start (Ampliwax, Perkin Elmer, Foster City, CA, USA). Primer sequences were as previously described (Shih *et al*, 2002). The PCR cycle was an initial step of 95°C for 3 min followed by 94°C for 1 min, 55°C for 1 min, 72°C for 1 min, then 25 cycles and a final cycle of 72°C for 8 min. Polymerase chain reaction products were subjected to electrophoresis in 2% agarose gels containing 0.2 *μ*g ml^−1^ ethidium bromide. Gels were visualised under UV light and photographed with Polaroid film (Polaroid Co., Cambridge, MA, USA). Photographs were scanned under direct light (Bio-Image, Millipore) for quantitation and illustration. Results of *p21* cDNA levels were normalised to *GADPH* cDNA levels from the same samples.

### Apoptosis/nucleosomes

Cells were harvested and washed twice with phosphate-buffered saline. Nucleosome ELISA assays were carried out according to the protocol provided by Oncogene Research Products (Cambridge, MA, USA) (Lin *et al*, 2002).

### Apoptosis/DNA fragmentation

Total genomic DNA was extracted according to the protocol of the SUICIDE-TRACK™ DNA LADDER ISOLATION KIT (Oncogene, Cambridge, MA, USA). DNA electrophoresis was performed in 1.5% agarose gels containing 0.2 *μ*g ml^−1^ of ethidium bromide. Gels were visualised under UV light and photographed with Polaroid film (Polaroid Co., Cambridge, MA, USA). Photographs were scanned under direct light (BioImage, Millipore) for quantitation and illustration.

## RESULTS

### Effects of resveratrol and a p53 inhibitor on MAPK activation, p53 phosphorylation and apoptosis in MCF-7 cells

Initial studies focused on the effects of resveratrol on phosphorylation, or activation, of MAPK (pERK1/2) and p53, and accumulation of these phosphorylated proteins in nuclear fractions of MCF-7 cells (Figure 1). Treatment of these cells with 1–100 *μ*M resveratrol for 4 h induced MAPK phosphorylation and serine phosphorylation of p53 at residues 15, 20 and 392. In addition, acetylation of p53 was induced by resveratrol treatment (Figure 1). Phosphorylation of ERK1 and ERK2, and of p53, resulted in translocation of these proteins to cell nuclei. The combined results from three experiments, normalised to a value of 1 in control samples, are shown in the graph of Figure 1.

In order to determine if resveratrol-induced apoptosis was p53 dependent, a specific p53 inhibitor, pifithrin-*α* (PFT-*α*) (Komarov *et al*, 1998), was added to cells in the presence or absence of resveratrol. Resveratrol-induced phosphorylation of serines 15, 20 and 392, and acetylation of p53, were inhibited by PFT-*α* (Figure 2A). In Figure 2B, the combined results of three nucleosome ELISA studies show a five-fold increase in nucleosome content in cells treated with resveratrol, and a reduction in the resveratrol effect with PFT-*α*. Increased nucleosome content is directly related to apoptosis (Lin *et al*, 2002; Shih *et al*, 2002). These findings indicate that resveratrol-induced apoptosis in MCF-7 cells is p53-dependent.

### Effect of oestradiol on resveratrol-induced MAPK activation, serine phosphorylation of p53 and apoptosis

In a 4 h study with MCF-7 cells, E_2_ (10^−9^ M) caused phosphorylation (activation) and nuclear translocation of MAPK, shown as pERK1 and pERK2 in Figure 3, lane 2. Furthermore, in cells treated with 10 *μ*M resveratrol for 4 h, 1–4 h of E_2_ exposure enhanced the activation and nuclear translocation of MAPK caused by resveratrol (lanes 4–7 compared with lane 3). In contrast to this enhancement by E_2_ of resveratrol-induced MAPK activation, the hormone inhibited resveratrol-induced serine phosphorylation of p53 in a time-dependent manner. Phosphorylation of serines 15, 20 and 392 by resveratrol, as well as acetylation of p53, were all progressively reduced in resveratrol-treated MCF-7 cells by the addition of E_2_ to resveratrol for 1–4 h.

In E_2_ concentration–response studies, there was hormone concentration-dependent enhancement of resveratrol-stimulated MAPK activation with 10^−11^–10^−9^ M E_2_ (Figure 4A, lanes 4–6) but reduction in resveratrol-induced phosphorylation of p53 serines 15, 20 and 392, and reduction in acetylation of p53 after treatment with both agents for 4 h (lanes 4–6). These latter effects were also E_2_ concentration-dependent. The effect of 10^−11^–10^−9^ M E_2_ on resveratrol-induced apoptosis in MCF-7 cells, indicated by nucleosome ELISA, is shown in Figure 4B. Dose-responsive inhibition by E_2_ of apoptosis in these resveratrol-treated cells correlates directly with the reduction in post-translational modification of p53 seen in Figure 4A, above.

### Effect of the oestrogen receptor-*α* (ER) inhibitor, ICI 182,780, on actions of resveratrol and oestradiol

E_2_ has been shown to block chemotherapy- or radiation-induced apoptosis through a plasma membrane receptor that appears to be the nuclear ER located in the cell membrane (Razandi *et al*, 2000). Therefore, we tested the E_2_ effect on resveratrol action in the presence of ICI 182,780 (ICI), an inhibitor of ER (Papaconstantinou *et al*, 2002). ICI 182,780 partially suppressed E_2_-induced ERK1/2 phosphorylation, or activation (Figure 5, comparing lanes 3 and 4). ICI 182,780 did not inhibit resveratrol-induced post-translational modifications of p53 (lane 5 *vs* lane 6). ICI 182,780 did enhance the effect of resveratrol on serine-15 phosphorylation of p53 (lane 6), although this effect was not statistically significant. E_2_ blocked serine phosphorylation and acetylation of p53 caused by resveratrol (lane 7), but in the presence of E_2_ and ICI, the inhibitory effect of the hormone on phosphorylation and acetylation of p53 was not seen (lane 8). In summary, signal transduction by resveratrol was enhanced by ICI, and the action on MAPK activation by E_2_ alone was inhibited by ICI. The inhibitory effect of E_2_ on resveratrol-induced phosphorylation and acetylation of p53 appears to be ER-mediated, in that this hormone effect was reversed by the ER inhibitor.

### Oestradiol inhibits resveratrol-induced p53 binding to DNA and p53-dependent gene expression

**Figure 6:**
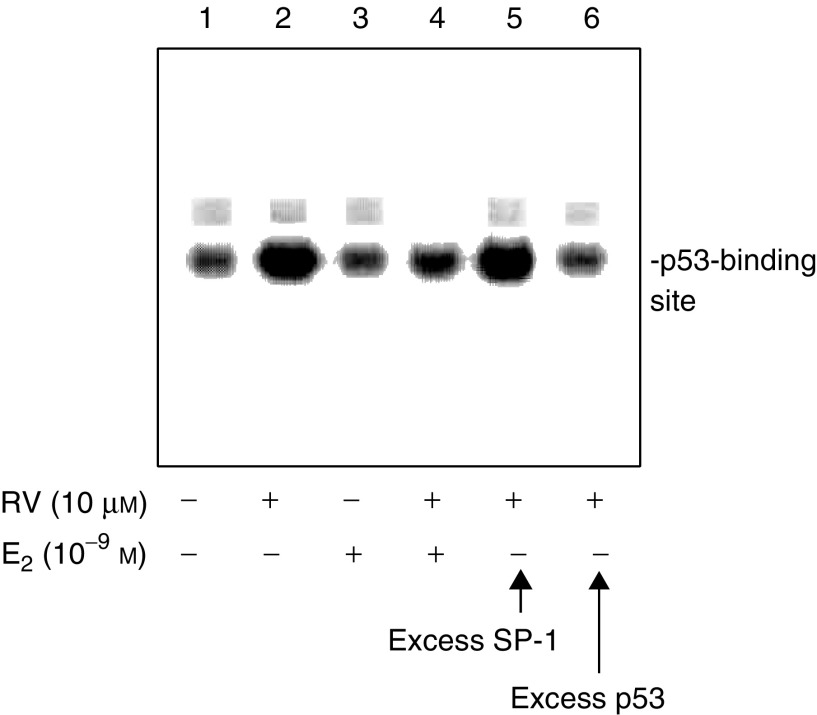
E_2_ inhibits resveratrol-induced p53 binding to a relevant oligonucleotide in an EMSA. The assay was carried out with nuclear fractions prepared after 4 h treatment of cells with 10 *μ*M resveratrol, 10^−9^ M E_2_, or both agents. p53 binding to the radiolabelled oligonucleotide was seen (lane 2). E_2_ inhibited p53 binding (lane 4 compared with lane 2). Excess unlabelled oligonucleotide (p53-oligo) decreased p53 binding to the labelled form (lane 6), whereas there was no change in p53 binding with an excess of unlabelled nonspecific SP-1 oligonucleotide (lane 5). This figure is representative of three similar experiments.

We studied the interaction of p53 with a p53-binding radiolabelled oligonucleotide added to nuclear extracts from resveratrol-treated cells, in the presence or absence of E_2_. Results shown in Figure 6

indicate that (1) resveratrol caused binding of p53 to the labelled oligonucleotide, and (2) E_2_ inhibited interaction of p53 and the oligonucleotide. Therefore, E_2_ not only inhibits resveratrol-induced post-translational modification of p53 as shown above in Figures 3, 4 and 5 but also inhibits DNA binding by p53.

**Figure 7:**
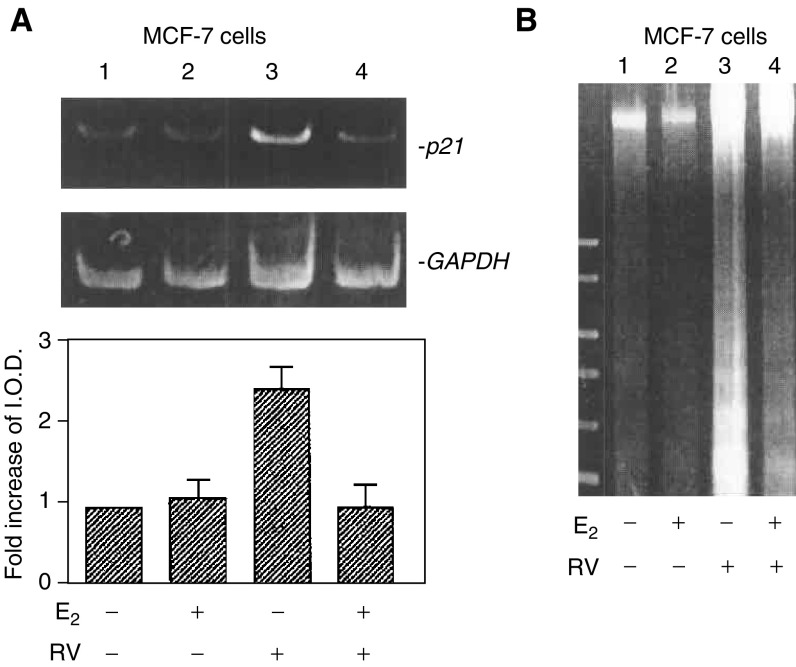
E_2_ inhibits p53-inducible *p21* expression and apoptosis caused by resveratrol. (**A**) MCF-7 cells were treated with 10 *μ*M resveratrol in the presence or absence of 10^−9^ M E_2_ for 24 h. Based on measurement of *p21* and *GAPDH* cDNA band densities (upper and lower images, respectively), and correction of *p21* densities for the levels of *GAPDH*, a 2.5-fold increase in *p21* cDNA abundance was seen with resveratrol treatment (lane 3 compared with lane 1). This increase in *p21* expression was significant (*P*<0.025) by analysis of variance. E_2_, 10^−9^ M, blocked *p21* transcription induced by resveratrol (lane 4 compared with lane 3, *P*<0.05), but had no effect in the absence of resveratrol. (**B**) MCF-7 cells were treated with 10 *μ*M resveratrol in the presence or absence of 10^−9^ M E_2_ for 24 h. Resveratrol caused DNA fragmentation, indicating apoptosis as shown in this representative figure (lane 3). This effect of resveratrol was inhibited by E_2_ (lane 4).

Resveratrol is known to induce p53-dependent *p21* expression in human thyroid cancer cells (Shih *et al*, 2002), an effect that is associated with apoptosis in these cells. Induction by resveratrol of *p21* expression in MCF-7 cells has been previously shown to lead to apoptosis (Pozo-Guisado *et al*, 2002). In our studies of transcription by RT–PCR in MCF-7 cells, we found that resveratrol caused transcription of *p21*, and that E_2_ inhibited this action of the stilbene (Figure 7A

). The biological end point of this effect of E_2_ was inhibition of resveratrol-induced apoptosis, as indicated by loss of DNA fragmentation (Figure 7B) and nucleosome ELISA (Figure 4B).

Oestradiol, 10^−9^ M, had no effect alone on *p21* expression in our experiments. Cariou *et al* (2000) have described a transient increase in *p21* content after 4 h treatment with 10^−8^ M E_2_ in MCF-7 cells, whereas in our studies, a lower concentration of hormone was used for 24 h. Others have reported that E_2_ can enhance abundance of p21 mRNA in D351 breast cancer cells (Levenson *et al*, 2003). The basis for these disparate effects of E_2_ is not clear, although differences in cell lines, in hormone concentration or in exposure time of cells to E_2_ (Levenson *et al*, 2003) may have contributed to the different results. The D351 cells used by Levenson and co-workers were MDA-MB-231 cells transfected with cDNA for *wt* ER, but the p53 in these cells is said to be nonfunctional (Toillon *et al*, 2002).

## DISCUSSION

Resveratrol, a phyto-oestrogen based on the fact that it binds to and activates ER, has similar affinity for ER*α* and ER*β*, but with 7000-fold lower affinity than E_2_ (Bowers *et al*, 2000). As has been shown for other selective ER modulators such as tamoxifen, resveratrol has been considered to have potential as an anti-breast cancer adjunct (Bhat *et al*, 2001). The stilbene has been shown to inhibit carcinogen-induced preneoplastic lesions and mammary tumours in rodent models (Bhat *et al*, 2001). In the present studies, treatment of MCF-7 breast cancer cells with 1–100 *μ*M resveratrol induced MAPK activation, serine phosphorylation and acetylation of p53 and p53-dependent apoptosis. In addition to these findings in breast cancer cells, we have previously shown that human thyroid and prostate cancer cells are susceptible to resveratrol-induced, p53-dependent apoptosis (Lin *et al*, 2002; Shih *et al*, 2002).

We have examined the possibility that the actions of resveratrol on MCF-7 cells might be modified by E_2_. Acting at the cell surface, oestradiol has been shown by several investigators to rapidly activate MAPK (Migliaccio *et al*, 1996; Razandi *et al*, 2003; Wade and Dorsa, 2003). We have confirmed this action of E_2_ in MCF-7 cells in this report (Figures 3, 4 and 5). Mitogen-activated protein kinase activation induced by oestrogen in breast cancer cells has been linked to cell proliferation but there have been no reports to indicate that MAPK activation in breast cancer cells is involved in apoptosis (Santen *et al*, 2002). In our studies, treatment of MCF-7 cells with 10 *μ*M resveratrol in the presence of 10^−9^ M E_2_ produced additive MAPK activation by E_2_ and resveratrol. However, E_2_ inhibited resveratrol-induced phosphorylation of serines 6, 15 and 392 in p53, as well as acetylation of p53, despite the enhancement by E_2_ of MAPK activity.

A downstream effect of this action of oestrogen on MAPK activation may be phosphorylation of Ser-118 of ER*α* and enhanced transcriptional activity of the receptor (Kato *et al*, 1995). We did not observe apoptosis as a downstream consequence of MAPK activation by E_2_ alone, compared with the actions of resveratrol. Thus, the confluence at MAPK of signals generated by resveratrol and E_2_ results, downstream of MAPK, in a divergence of effects on nuclear transcription by p53 and by E_2_. This is consistent with the existence of discrete intracellular pools of MAPK (ERK1/2) separately regulated by resveratrol and E_2_.

The antiapoptotic effect of E_2_ in the presence of resveratrol involves rapid stimulation of signalling cascades, including the ERK1/2 pathway. In our studies, the ER inhibitor ICI 182,780 suppressed both E_2_-induced MAPK activation and the inhibitory effect of E_2_ on resveratrol-induced serine phosphorylation of p53. Although resveratrol has been shown by others to have oestrogen-like activity in a transcriptional assay in cells expressing predominantly ER*α* (Bhat *et al*, 2001), we found that ICI 182,780, in a relatively low concentration of 3 nM, did not diminish resveratrol-induced activation of MAPK and serine phosphorylation of p53, and in fact appeared to enhance these effects of the stilbene. The concentration we used was similar to the inhibitory 10 nM concentration used in MCF-7 cells by Cariou *et al* (2000).

Others have also reported that activities of resveratrol are not affected by ICI 182,780 (Cho *et al*, 2002). Since E_2_ action was inhibited by ICI 182,780 in our studies, while resveratrol activity was minimally affected by ICI 182,780, the inhibitory effect of E_2_ on resveratrol-induced serine phosphorylation of p53 was interpreted to be ER mediated. Studies by others have shown that ICI 182,780 may enhance the effects of doxorubicin, paclitaxol and tumour necrosis factor-*α* on the production of apoptosis in MCF-7 cells (Burow *et al*, 2001).

Oestradiol alone had no effect on the binding of p53 to a relevant oligonucleotide. However, E_2_ did inhibit resveratrol-induced binding of p53 to the oligonucleotide (Figure 6). This finding is consistent with E_2_ inhibition of post-translational modifications of p53 that are essential for p53-dependent gene transcription. These results have implications for the possible use of resveratrol therapeutically in cancer treatment, as they suggest that resveratrol may be most effective in an oestrogen-depleted environment.

The transcriptional activity of p53 is subject to modulation by other hormones as well. For example, glucocorticoids prevent p53-induced apoptosis through interaction of the glucocorticoid receptor (GR) with p53 and the E3 ubiquitin ligase, Hdm2 (Sengupta and Wasylyk, 2001) in cytosol. Another example of a modulator is the mdm2 gene that is positively regulated by p53, yet the mdm2 protein binds p53, forming a feedback loop that controls mdm2 activity (Qi *et al*, 1999).

Since the effect of resveratrol that we have demonstrated in MCF-7 cells centres on the modification and action of the tumour suppressor p53, it is conceivable that cancer cells harbouring mutant p53 might not respond in the same manner. However, studies by Hsieh *et al* (1999) have documented resveratrol-induced reduction in growth of MDA-MB-435 cells, which contain a mutant p53. In our studies of the actions of resveratrol on DU145 prostate cancer cells, which also contain mutant p53 (Zhang *et al*, 2003), resveratrol did induce apoptosis. Future studies are to be directed towards the identification of critical sites on p53 for resveratrol action.

The present studies indicate that ambient E_2_ may antagonise actions of resveratrol in selected treatment paradigms. Our results suggest the complexity of actions of resveratrol in both *in vivo* and *in vitro* settings. Were resveratrol to be tested in intact animals, it would be important to control for levels of oestrogen, and useful to examine actions of the stilbene in the presence of an E_2_ antagonist.
